# Sacrocolpopexy: Patient Outcomes Support the Use of Non-Crosslinked Acellular Dermal Matrix as an Alternative to the Synthetic Polypropylene Mesh

**DOI:** 10.1089/gyn.2019.0010

**Published:** 2019-12-09

**Authors:** Magdalene Karon, Somu Chatterjee

**Affiliations:** Department of Obstetrics/Gynecology, Women's Hospital Saint Joseph East, Dr. Karon's Pelvic Reconstructive Surgery & Research Center, Lexington, KY.

**Keywords:** sacrocolpopexy, non-crosslinked acellular matrix, viable cryopreserved umbilical tissue, regenerative healing, mesenchymal stem cells

## Abstract

***Objective:*** This study was conducted to evaluate the use of non-crosslinked acellular dermal matrix (ADM) in laparoscopic sacrocolpopexy by analyzing clinical outcomes and patient-satisfaction surveys.

***Materials and Methods:*** Two hundred and eleven patients underwent laparoscopic sacrocolpopexy for pelvic organ prolapse (POP) between January 6, 2012, and December 31, 2017. Each patient had her pelvic-floor measurements diagrammed with the POP-Q [Quantification] system using the interactive tool provided by the American Urogynecologic Society. The Pelvic Floor Distress Inventory (PFDI-20) and Pelvic Floor Impact Questionnaire (PFIQ-7), used to make comprehensive assessments of women with urogynecologic problems, were mailed to all patients. If responses were not received, the patients were contacted by telephone. Patients who were not reached by either mail or telephone had their charts individually reviewed to extract the information. Trained surveyors scored the PFDI-20 and the PFIQ-7 questionnaires. The de-identified data were analyzed for patient satisfaction and outcomes. This information was obtained by a review of patient charts at 4-week postoperative and annual examinations; any phone calls with complaints and/or problem office visits were noted. Biopsies from the sacrocolpopexy area were taken if a patient had another incidental gynecologic procedure unrelated to the prolapse or at the time of repeat sacrocolpopexy for POP and the paraffin cell block was sent to McGowan Institute for Tissue Regeneration.

***Results:*** One hundred and five patients responded to the survey. Charts were completed for 106. The majority of interviewed patients stated that they were doing a “little better” or “much better” (77/88; 87.5%). The third-quartile PFDI-20 score was 93 with a median of 60 and the PFIQ-7 score was 43 with a median of 29. Five patients underwent reoperations (4.76%). The most-common postoperative complaint was overactive bladder symptoms, followed by vaginal discharge. Histology showed either a lack of regenerative healing tissue at the failure site or good results showing neovascularization and a presence of connective and ligamentous tissue around the matrix. No intense fibrosis or neoplastic formation was reported.

***Conclusions:*** A non-crosslinked ADM patch can be a good alternative to synthetic polypropylene mesh in patients undergoing sacrocolpopexy for POP.

## Introduction

This article is about a follow-up study to the original one published in the *Journal of Gynecologic Surgery* in 2017, by the current author, entitled “Sacrocolpopexy: A Modification of the Standard Laparoscopic Procedure to Adopt *[sic]* to the Properties of a Biologic Matrix Patch.”^1^ Previous results showed a 9-patient postoperative Pelvic Floor Distress inventory (PFDI-20) average of 25.47 ± 29.68. Patient-reported satisfaction with postsurgical outcomes ranged from 70% to 100%. The current study evaluated clinical outcomes, satisfaction, and postoperative events in patients who underwent sacrocolpopexy with non-crosslinked acellular dermal matrix (ADM) between the years 2012 and 2017.

Sacrocolpopexy is considered the “gold standard” procedure for pelvic organ prolapse (POP). By aligning the original axis of the vagina and providing a strong apical support, sacrocolpopexy restores the original anatomical position before the POP occurred. Open sacrocolpopexy via a laparotomy has been performed successfully for many years but was considered more invasive than the transvaginal approaches.

Advanced laparoscopy and optics have made this procedure an outpatient/23-hour observation operation with faster recovery and all the other advantages of less-invasive surgery. This also applies to repeat laparoscopic procedures that are generally less-challenging than an open laparotomy. A repeat laparoscopic procedure is less-challenging after a previous laparoscopy rather than previous open laparotomy. Adhesions and distortion of anatomy are more common after an open procedure. Bowel adhesions under the incision site, in particular, make the placement of laparoscopic ports more difficult.

Different graft materials have been used but polypropylene mesh has dominated the market for the last 10 years and is the main material used in sacrocolpopexy. Cadaveric fascia has been used as an alternative but is not considered as strong of a support. Acellular cadaveric fascia-lata and dermal matrix patches have entered the market and are being investigated. POP may reoccur after sacrocolpopexy with polypropylene mesh, and it is estimated that up to 18% of women are expected to undergo reoperation.^2^ Both patients and surgeons are aware of the litigations surrounding polypropylene-mesh complications of erosion, migration, and failures. However, until now, a better alternative has not been available.^3–7^ The effectiveness of a biologic patch has been the subject of much debate.^8^

The current author has data collected from June 2012 to December 2017 on patients, each of whom underwent laparoscopic sacrocolpopopexy with a biologic patch consisting of a non-crosslinked ADM (Life Cell now under the ownership of Allergan). The product manufacturer did not recommend cutting the patch and resuturing it into a Y-shaped graft, fearing that this would create a point of weakness and potential separation leading to a failure of the suspension. Therefore, the technique was modified as described in the current author's previous publication.^1^ An important component of the modified technique involved incorporation of the round ligaments for lateral support when possible. One single piece of a matrix patch was used. To complement each patient's patients' individual anatomy the bottom 2 cm (± 1 cm) were split in the middle and anchored to prevent too much of an anterior pull. A wide attachment base to the vaginal cuff or cervix is important to spread the tension of the lower attachment of the patch. Eight or more permanent interrupted sutures were used the Gore-Tex sutures were chosen because of their pliability to help prevent tissue from tearing away from the suture when traction was present.

The ADM has been approved by the U.S. Food and Drug Administration for use as a

[s]oft tissue patch to reinforce soft tissue where weakness exists and for surgical repair of damaged or ruptured soft tissue membranes. Indications for use include the repair of hernias and/or body wall defects which require the use of reinforcing or bridging material to obtain the desired surgical outcome during open or laparoscopic procedures.^9^

Thus, ADM has an on-label use in sacrocolpopexy just as synthetic polypropylene mesh does. Other specialties have adopted ADM for procedures such as hernia repairs and breast cancer reconstruction.

The objective of the current research was to evaluate the use of non-crosslinked ADM in laparoscopic sacrocolpopexy through the analysis of clinical outcomes and patient-satisfaction surveys, the Pelvic Floor Distress Inventory (PFDI-20) and Pelvic Floor Impact Questionnaire (PFIQ-7).^10^

## Materials and Methods

### Design

A retrospective survey of postoperative sacrocolpopexy patients was performed between April and June of 2018 by trained surveyors from the University of Kentucky Physician's Assistant Studies. The PFDI-20 and PFIQ-7 have been used as validated tools for assessment of patient symptoms with POP in the field of urogynecology. These questionnaires were mailed to all postoperative patients who had surgery performed between June 2012 and December 2016. Patients who did not return the questionnaires were contacted by telephone and data were collected from them. Patients who could not be reached via mail or telephone or were operated on in 2017 were assessed by chart review. The surveyors were blinded to the data. The data analysis was conducted on de-identified data. The primary investigator, the current author, MD individually assessed the patients with outlier scores; one of these patients did not understand English well and the other patient was 80 years old and had been diagnosed with dementia.

Institutional review board approval (IRB) for this study was obtained from the University of Louisville School of Medicine in Louisville, KY.

### Surgical technique

The complete surgical technique was described in the current author's previous publication.^1^ Prior to 2012, the surgical technique was started with a patch from a porcine bladder source. However, the uni-sided orientation of the matrix patch (ACell), difficult handling during suturing, and the patch's absorptive properties made it less-desirable than the dermal source of matrix used in the current study. The ADM was secured to the vaginal apex with Gore-Tex^®^ (W.L. Gore & Associates, Inc.) interrupted sutures. The sutures were spaced so that as much of the vaginal anterior and posterior attachment possible would be provided to serve as a wide base. For the lateral support, the round ligaments were incorporated if they were identifiable. Sacral attachment was also performed with GORE-TEX sutures. Care was taken to use the anterior ligament for fixation and not to drive the sutures too deep into the intervertebral disc annulus. Peritoneal closure over the graft is important so that the entire sacrocolpopexy is in the retroperitoneal space at the end of the procedure.

### Follow-up

All patients were examined 4–6 weeks postoperatively. Patients' POP-Q [Quantification] interactive computer assessments and scores were compared to their preoperative diagrams. Patients were next examined at their annual well-woman examinations or as necessary for other complaints.

### Questionnaire/ survey on postoperative quality of life

The condition-specific quality of life PFDI-20 and PFIQ-7 questionnaires were completed by the patients or by the trained surveyors who contacted the patients. Missing data were substituted, using the scores as directed in the PFDI-20 and PFIQ-7. The de-identified PFDI-20 and PFIQ-7 data were analyzed for patient satisfaction. For patients who could not be reached by telephone, did not return questionnaires, or had been operated on in 2017, the charts were individually reviewed to note any postoperative complaints. Additionally, patients' symptoms and examiners' physical findings were compiled from patients' office visits or their annual routine well woman examinations.

In total, 106 patients (operated on 2012–2017) who did not fill out the survey (*n* = 105) ; those cases were subjected to complete chart review. This included patients who had 4-week postoperative visits, visits for call-back complaints, any postoperative patient operated on between 2012 and 2017 scheduled for an annual follow-up with/without associated symptoms, and anyone who could not be reached by telephone or mail to complete questionnaire for 2012–2016. The patient groups in chart review were not mutually exclusive to specific symptoms (i.e., the same patient may have multiple complaints/symptoms; Table 1). 

**Table 1.** Post-Sacrocolpopexy Follow-Up Time (in Months)

Months	Number of patients who reported for follow-up
7–12 mo	1
13–24 mo	13
25–36 mo	24
37–60 mo	61
Other >60 mo	1

mo, months.

### Histology

Biopsies of the distal and proximal ends of the sacrocolpopexy were collected if a patient underwent another pelvic operation unrelated to the prolapse or if the prolapse reoccurred and the sacrocolpopexy was repeated. The paraffin cell blocks from the biopsies were sent to Stephen Badylak, MD, PhD, DVM at the McGowan Institute for Regenerative Medicine, a part of the University of Pittsburgh. The techniques for tissue processing and slide preparation are parts of the institute's team research and are not subjects of this article. The McGowan Institute was consulted on “fee-per-service” basis. The pathologists were blinded to which patient's sample was submitted for tissue assessment. Examples of the slide illustrations were published in the original article.^1^

## Results

### Patient demographics

Two hundred and eleven patients underwent laparoscopic sacrocolpopexy between January 6, 2012, and December 31, 2017, with the biologic ADM. The mean age of patients that responded to the questionnaire was 56.49 ± 14 years. Patients' morbidities included truncal obesity, diabetes, cardiovascular conditions, and smoking. Ethnicity and social status of the patient population reflected a cross-section of urban, suburban, and some rural patients. The clinical setting was a teaching hospital but not a university hospital.

### Patient satisfaction

Of the interviewed patients, 85.22% (*n* = 75/88) reported the treatment of their prolapse as “very successful” (57.95%; *n* = 51) or “moderately successful” (27.27%; *n* = 24). Another 9.09% (*n* = 8/88) reported the treatment as “somewhat successful, and only 5.68% (*n* = 5/88) reported the treatment was “not at all successful.” Similarly, 88.5% (*n* = 77) of interviewed patients reported their current health status as a “little better” (16.09%; *n* = 14) or “much better” (72.42%; *n* = 63), compared to how they were doing before the pelvic-floor operation. Another 6.9% (*n* = 6) reported they were “about the same” and 4% (*n* = 4) reported that their health status was worse compared to their preoperational status.

### Clinical outcomes

**FIG. 1.**
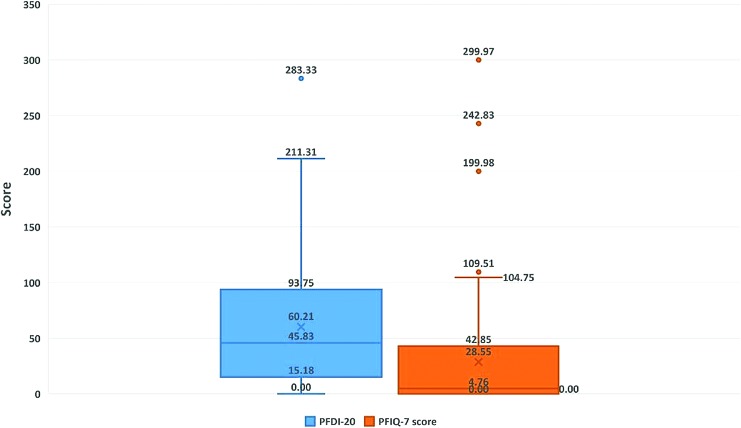
Box plots distribution of Pelvic Floor Distress Inventory (PFDI-20) and Pelvic Floor Impact Questionnaire (PFIQ-7) score quartiles on follow-up of post-sacrocolpopexy patients receiving the biologic matrix (*n* = 105). Color images are available online.

The PFDI-20 and PFIQ-7 scores and their components are listed in Table 2. The box plots (Fig. 1) visualize the distribution of the PFDI-20 and PFIQ-7 scores, while nullifying the effect of the outliers on the mean. The mean PFIQ-7 score was 28.55 with a median of 4.76, and a first-quartile score of 0 and a third quartile score of 42.85.

**Table 2.** Average Scores of PFDI-20 & PFIQ-7 Components in Biological Matrix Post-Sacrocolpopexy Patients (*N* = 105)

Instruments	Scores
POPDI-6	16.94
CRADI-8	15.88
UDI-6	27.38
PFDI-20^**^	60.21
UIQ-7	13.76
CRAIQ-7	8.34
POPIQ-7	6.44
PFIQ-7^*^	28.55

PFDI-20, Pelvic Floor Distress Inventory–20; PFIQ-7, Pelvic Floor Impact Questionnaire–7; POPDI-6, Pelvic Organ Prolapse Distress Inventory–6; CRADI-8, Colorectal–Anal Distress Inventory–8; UDI-6, Urinary Distress Inventory–6; UIQ-7, Urinary Impact Questionnaire–7; CRAIQ-7, Colorectal–Anal Impact Questionnaire–7; POPIQ-7; Pelvic Organ Prolapse Impact Questionnaire–7.

^*^ Pelvic Floor Impact Questionnaire (PFIQ-7) is a sum of UIQ-7, CRAIQ-7 and POPIQ-7.

^**^ Pelvic Floor Distress Inventory (PFDI-20) is a sum of POPDI-6, CRADI-8 and UDI-6.

### Frequent postoperative events

**FIG. 2.**
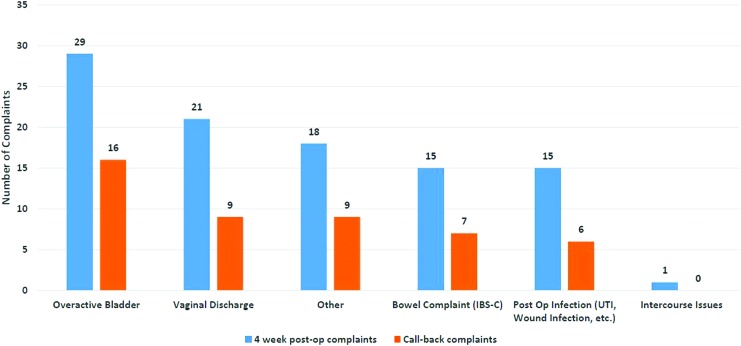
Most common symptom complaints/frequency of symptoms in post-sacrocolpopexy patients at 4 postoperative (postop) weeks versus any subsequent call-back visits (*n* = 106). IBS-C, irritable bowel syndrome with constipation; UTI, urinary-tract infection. Color images are available online.

**FIG. 3.**
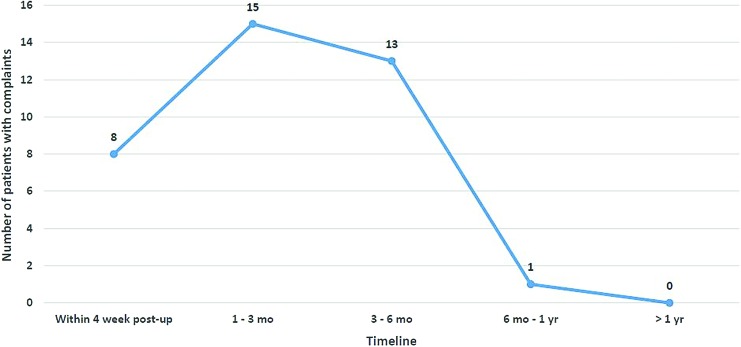
Timeline of call-back complaints. The number of call-back complaints increased during the first 3 postoperative (postop) months and then dramatically declined after 3 months up to 1 year post-sacrocolpopexy. mo, months; yr, year. Color images are available online.

A chart review of 106 patients revealed that overactive bladder (OAB) symptoms of urgency and frequency was the most commonly reported event and reason for a postoperative visit within the first postoperative month. This was followed by vaginal discharge (Fig. 2). The number of call-back complaints increased during the first 3 postoperative months and then dramatically declined after 3 months up to 1 year post-sacrocolpopexy (Fig. 3).

### Intraoperative findings during repeat sacrocolpopexy

The reoperation rate was 5/211 (2.37%). The timeframe of recurrence varied from 13 months to 36 months. Visual inspection showed that, in each of these patients, the pelvis was clear of adhesions, the anatomy was clear, and the previous sacrocolpopexy placement was identified easily. There was no dense scar tissue anywhere. The vaginal apex was flexible to manipulation from below, making the new reattachment of the ADM more flexible. The anatomical structures, such as the ureters, the bladder, and the colorectal area were not distorted by scarring or masked by adhesions.

**FIG. 4.**
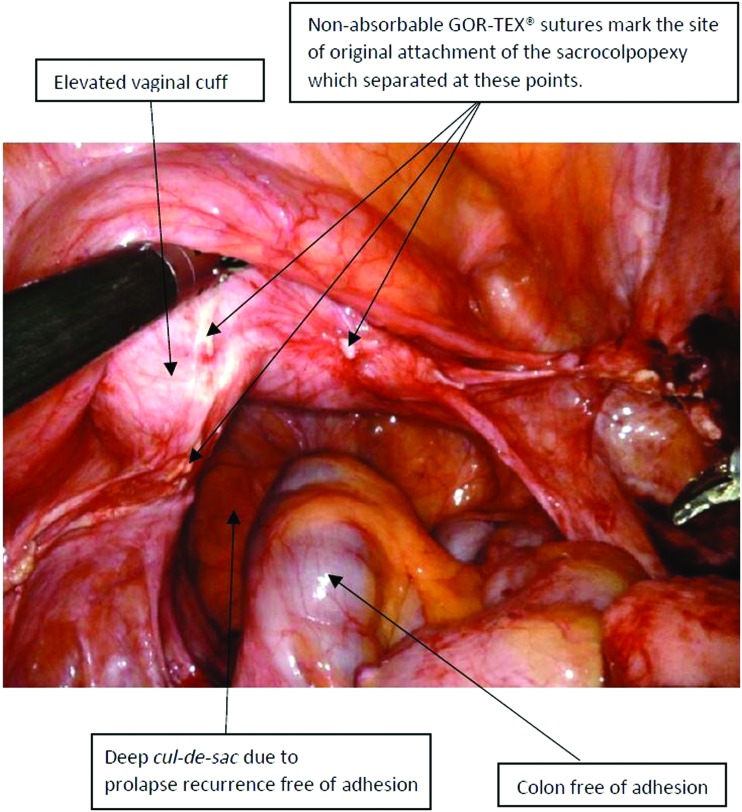
Preoperative field view in a patient with sacrocolpopexy with acellular dermal-matrix patch presenting for reoperation after prolapse recurrence. Color images are available online.

**FIG. 5.**
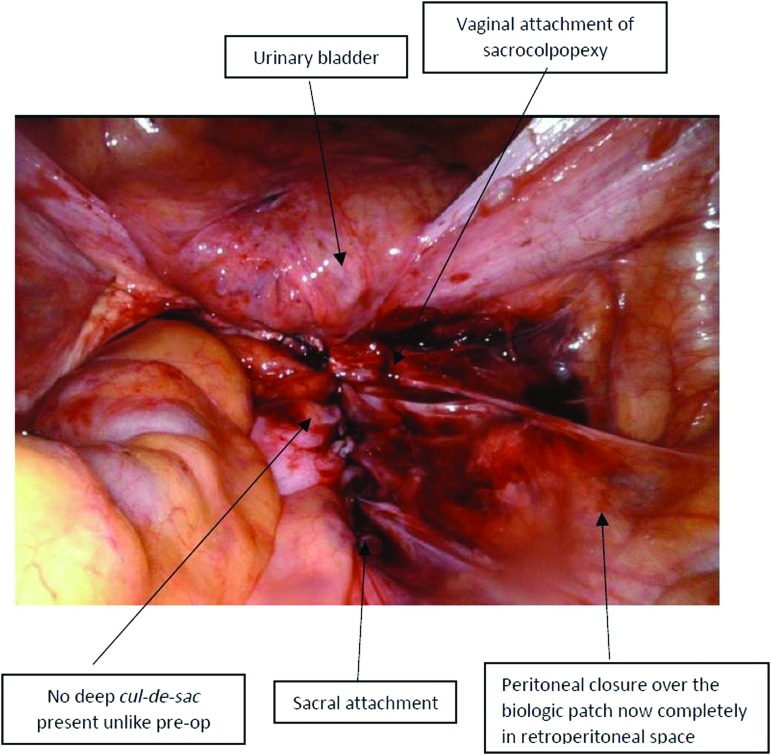
Postoperative field view in a post-sacrocolpopexy patient with acellular dermal-matrix patch after correction of prolapse recurrence. preop, preoperative. Color images are available online.

Importantly, there was no old failed permanent mesh to resect, which can be quite challenging to separate from the structures to which it is attached (it is never found “free floating”) as the principles of polypropylene mesh healing is by scar-tissue formation. The nonabsorbable GORE-TEX sutures served as a good marker to identify both the proximal and the distal attachment of the previous sacrocolpopexy. The point of failure or separation of the patch was always at the lower (vaginal) end and not at the sacral attachment (Fig. 4). Post-operative comparison (Fig. 5) shows the peritoneal closure over the biologic patch now completely in retroperitoneal space.

### Histology

Biopsies from the failed vaginal attachment area of the patch showed no “foreign body” presence, as the ADM patch was absorbed without scar formation from an inflammatory response as is seen with the synthetic polypropylene mesh. No evidence of tissue regeneration was found, which may help to explain the reason for the suspension failure. Conversely, biopsies from the sacral attachment area of the sacrocolpopexy ADM patch, as reported by the McGowan Institute, showed excellent neovascularization, and revealed the presence of collagen and ligamentous tissue that was laid down within and next to the matrix as occurs in the process of regenerative healing. There was no seroma formation or liquefaction of tissue as was observed with a previously used product made from a porcine bladder origin (ACell).

Given that these biopsies were not taken in the early phases of regenerative healing but more than 1 year later, when the prolapse reoccurred, macrophage infiltration was no longer demonstrable on the pathology slides. Importantly, the pathology department never reported any neoplastic growth in any of these biopsies. All of the biopsies were performed laparoscopically and not transvaginally at the time of an incidental operation.

It was felt that asymptomatic patients for an elective vaginal biopsies would not be compliant with the IRB requirements for this study, as it would inconvenience the patient to do a medically not indicated procedure.

## Discussion

The favorable patient outcomes in this study support the use of ADM as an alternative to synthetic polypropylene mesh in sacrocolpopexy for the correction of POP. This study was preceded only by 1 other study using a biologic non-crosslinked acellular matrix in sacrocolpopexy done on primates by Rui Liang, MD, and Pamela Maolli, MD, PhD.^3,11,12^ These investigators were able to show that the acellular matrix (ACell) attenuated the negative impact on the vaginal flora of the polypropylene mesh. The McGowan Institute for Regenerative Healing also processed the tissue slides for the abovementioned studies.

The PFDI-20 and PFIQ-7 scores (Table 2) were comparable to the post-sacrocolpopexy and post–pelvic-surgery scores found in the literature.^13–22^ Patients reported a feeling of overall improvement. The OAB symptom complaints comprised the most-common call-back reason for up to 3 months postoperatively and then steadily declined up to 1 year (Fig. 3). The OAB symptoms were treated and patients responded well to the oral medications indicated for that diagnosis. The postoperative Urinary Distress Inventory–6 total score was similar to those in other studies.^13,22,23^ The reoperation rate was 2.37%, which was similar to other studies in the literature.^24–27^ In all 5 cases, the pelvis was clear of adhesions with the added advantage of not having to remove an old mesh. The operative time required for the repeat sacrocolpopexy was about the same to ∼30 minutes longer, and most of the time it was possible to use the same laparoscopic incisions.

To give patients who underwent repeat surgeries an advantage of better healing after that second surgery, cryopreserved umbilical tissue with viable cells (vCUT) was added to the biologic non-crosslinked acellular matrix at each end of the new sacrocolpopexy reattachment. There are commercially available human allograft vCUT products.

The principle behind the use of vCUT is the added advantage of providing the pluripotent mesenchymal life stem cells with the growth factors and antimicrobial factors that are also present. The results and outcomes of complementing these two products will be reported in a separate publication. The added vCUT is only mentioned here because it was used to help repair and complement the tissues in the failed cases instead of repeating the same operation and product. There is collagen matrix present in the Warton's umbilical jelly, but the “scaffold” matrix was already present with a stronger structure in the biologic ADM.

When ADM is used alone, it has to rely on the patient's healthy immune system to provide a healing response. Patients' own stem cells and macrophages need to be attracted to the matrix to start the regenerative process within the matrix and next to it. However, if a patient is immunocompromised, has diabetes, or is a smoker, she will not have enough healthy healing cells or stem cells of her own. This is why platelet-rich plasma (PRP) has not been effective for these patients. No reports were found regarding successful use of PRP in the female pelvis. With the advancement in the technology and research it is hoped that biologic patches will be improved, as other specialties are using them for other purposes. Future studies are needed to investigate other options to the polypropylene mesh, especially in recurrent POP.

It is especially interesting that vCUT and other similar products of placental/umbilical origin, which are considered human allografts, are now regulated by the FDA under 21 CFR, part 1271, 3(d)(1) and section 361, covering human cells, tissues, and cellular/tissue-based products.^28^ There is a multispecialty indication released by the FDA for the use of these products until December of 2020, as long as they are used in a homologous and minimally manipulated way.^28^ The female pelvis provides that homologous environment. This is an ongoing study that will be reported at a later date in a separate article.

### Study limitations

The retrospective design of this study limited the ability to assess the improvement in PFDI-20 and PFIQ-7 scores between the pre- and postoperative status. A randomized controlled trial might add more information regarding the current results.

### Study strengths

The number of patients (211) who underwent sacrocolpopexy, with the length of the study being more 5½ years, makes it the largest study to date in this category.

## Conclusions

The favorable patient outcomes in this study, combined with the results comparable to the ones reported with polypropylene mesh but without the erosion complications, makes the ADM biologic patch a good alternative in laparoscopic sacrocolpopexy.

## References

[1] Karon M Sacrocolpopexy: A modification of the standard laparoscopic procedure to adopt *[sic]* it to the properties of a biologic matrix patch. J Gynecol Surg 2017;33:248

[2] Arsene E, Giraudet G, Lucot JP, Rubod C, Cosson M Sacral colpopexy: Long-term mesh complications requiring reoperation(s). Int Urogynecol J 2015;26:353 25323309 10.1007/s00192-014-2514-7

[3] Liang R, Zong W, Palcsey S, Abramowitch S, Moalli PA Impact of prolapse meshes on the metabolism of vaginal extracellular matrix in rhesus macaque. Am J Obstet Gynecol 2015; 212:174.e1 25128444 10.1016/j.ajog.2014.08.008 PMC4312539

[4] Jallah Z, Liang R, Feola A, et al. The impact of prolapse mesh on vaginal smooth muscle structure and function. BJOG 2016;123:1076 26301457 10.1111/1471-0528.13514 PMC5201168

[5] Akyol A, Akca A, Ulker V, et al. Additional surgical risk factors and patient characteristics for mesh erosion after abdominal sacrocolpopexy. J Obstet Gynaecol Res 2014;40:1368 24754851 10.1111/jog.12363

[6] Ellington DR, Richter HE Indications, contraindications, and complications of mesh in surgical treatment of pelvic organ prolapse. Clin Obstet Gynecol 2013;56:276 23563869 10.1097/GRF.0b013e318282f2e8 PMC3644007

[7] Dallenbach P To mesh or not to mesh: A review of pelvic organ reconstructive surgery. Int J Womens Health 2015;7:331 25848324 10.2147/IJWH.S71236 PMC4386830

[8] Yurteri-Kaplan LA, Gutman RE The use of biological materials in urogynecologic reconstruction: A systematic review. Plast Reconstr Surg 2012;130(5[suppl2]):242S 23096979 10.1097/PRS.0b013e31826154e4

[9] U.S. Food and Drug Administration. 510(k) Summary: LTM–Laparoscopic Surgical Mesh. 4 17, 2013 Online document at: www.accessdata.fda.gov/cdrh_docs/pdf13/K130817.pdf Accessed 91, 2019

[10] Barber MD, Chen Z, Lukacz E, et al. Further validation of the short form versions of the Pelvic Floor Distress Inventory (PFDI) and Pelvic Floor Impact Questionnaire (PFIQ). Neurourol Urodyn 2011;30:541 21344495 10.1002/nau.20934 PMC3759146

[11] Liang R, Knight K, Barone W, Powers RW, Nolfi A, Palcsey S, Abramowitch S, Moalli PA Extracellular matrix regenerative graft attenuates the negative impact of polypropylene prolapse mesh on vagina in rhesus macaque. Am J Obstet Gynecol 2017;216:153.e1 27615441 10.1016/j.ajog.2016.09.073 PMC5290183

[12] Liang R, Knight K, Easley D, Palcsey S, Abramowitch S, Moalli PA Towards rebuilding vaginal support utilizing an extracellular matrix bioscaffold. Acta Biomater 2017;57:324 28487243 10.1016/j.actbio.2017.05.015 PMC5639927

[13] Houlihan S, Koenig N, Friedman B, Lee T, Geoffrion R Fibroid surgery and improvement in bladder symptoms: The FAB study. Neurourol Urodyn 2018;37:1965 29862556 10.1002/nau.23541

[14] Gutman RE, Ford DE, Quiroz LH, Shippey SH, Handa VL Is there a pelvic organ prolapse threshold that predicts pelvic floor symptoms? Am J Obstet Gynecol 2008;199:683.e1 18828990 10.1016/j.ajog.2008.07.028 PMC2705877

[15] Nosti PA, Mcdermott CD, Schilder JM, Stehman FB, Woodman PJ Symptoms of pelvic floor disorders and quality of life measures in postoperative patients with endometrial cancer. Clin Ovarian Other Gynecol Cancer 2012;5:27

[16] Wiegersma M, Panman CM, Kollen BJ, et al. Pelvic floor muscle training versus watchful waiting or pessary treatment for pelvic organ prolapse (POPPS): Design and participant baseline characteristics of two parallel pragmatic randomized controlled trials in primary care. Maturitas 2014;77:168 24268876 10.1016/j.maturitas.2013.10.014

[17] Letouzey V, Mercier G, Adjoussou S, Bohoussou E, Mares P, de Tayrac R Can the PFDI (Pelvic Floor Distress Inventory) or PFIQ (Pelvic Floor Impact Questionnaires) be used to predict outcome in pelvic reconstructive surgery? Prog Urol 2013;23:940 24010925 10.1016/j.purol.2013.04.010

[18] Abdullah B, Nomura J, Moriyama S, Huang T, Tokiwa S, Togo M Clinical and urodynamic assessment in patients with pelvic organ prolapse before and after laparoscopic sacrocolpopexy. Int Urogynecol J 2017;28:1543 28283710 10.1007/s00192-017-3306-7

[19] Chevrot A, Droupy S, Linares E, de Tayrac R, Costa P, Wagner L Impact of laparoscopic sacrocolpopexy on symptoms, health-related quality of life and sexuality: A 3-year prospective study [in French]. Prog Urol 2016;26:558 27052819 10.1016/j.purol.2016.02.009

[20] Culligan PJ, Gurshumov E, Lewis C, Priestly JL, Komar J, Shah N, Salamon CG Subjective and objective results 1 year after robotic sacrocolpopexy using a lightweight Y-mesh. Int Urogynecol J 2014;25:731 24264283 10.1007/s00192-013-2265-x PMC4544463

[21] Laas E, Haddad M, Muhlstein J, Bendifallah S, Ballester M, Darai E Preoperative quality of life questionnaires are an adequate tool *[sic]* to select women with genital prolapse for laparoscopic sacrocolpopexy. Int Urogynecol J 2017;28:1833 28725910 10.1007/s00192-017-3423-3

[22] Thibault F, Costa P, Thanigasalam R, et al. Impact of laparoscopic sacrocolpopexy on symptoms, health-related quality of life and sexuality: A medium-term analysis. BJU Int 2013;112:1143 24007194 10.1111/bju.12286

[23] Salerno J, de Tayrac R, Droupy S, Costa P, Llinares E, Fatton B, Wagner L Impact of laparoscopic sacrocolpopexy, with or without a midurethral sling, on lower urinary tract symptoms [in French]. Prog Urol 2016;26:401 27068055 10.1016/j.purol.2016.03.003

[24] Linder BJ, Occhino JA, Habermann EB, Glasgow AE, Bews KA, Gershman B A national contemporary analysis of perioperative outcomes of open versus minimally invasive sacrocolpopexy. J Urol 2018;200:862 29630983 10.1016/j.juro.2018.03.131

[25] Mueller MG, Jacobs KM, Mueller ER, Abernethy MG, Kenton KS Outcomes in 450 women after minimally invasive abdominal sacrocolpopexy for pelvic organ prolapse. Female Pelvic Med Reconstr Surg 2016;22:267 27054799 10.1097/SPV.0000000000000269

[26] Orhan A, Ozerkan K, Vuruskan H, Ocakoglu G, Kasapoglu I, Koşan B, Uncu G Long-term follow-up of laparoscopic sacrocolpopexy: Comparison of two different techniques used in urology and gynecology. Int Urogynecol J 2019;30:623 30627828 10.1007/s00192-018-03858-w

[27] Diwadkar GB, Barber MD, Feiner B, Maher C, Jelovsek JE Complication and reoperation rates after apical vaginal prolapse surgical repair: A systematic review. Obstet Gynecol 2009;113(2[pt1]):367 19155908 10.1097/AOG.0b013e318195888d

[28] U.S. Food and Drug Administration (FDA). FDA Regulation of Human Cells, Tissues, and Cellular and Tissue-Based Products (HCT/P's) Product list. Last updated 2 1, 2018 Online document at: www.fda.gov/vaccines-blood-biologics/tissue-tissue-products/fda-regulation-human-cells-tissues-and-cellular-and-tissue-based-products-hctps-product-list Accessed 91, 2019

